# Integration of clinical demographics and routine laboratory analysis parameters for early prediction of gestational diabetes mellitus in the Chinese population

**DOI:** 10.3389/fendo.2023.1216832

**Published:** 2023-10-13

**Authors:** Hesong Zhang, Juhua Dai, Wei Zhang, Xinping Sun, Yujing Sun, Lu Wang, Hongwei Li, Jie Zhang

**Affiliations:** ^1^ Department of Clinical Laboratory, Peking University International Hospital, Beijing, China; ^2^ State Key Laboratory of Molecular Vaccinology and Molecular Diagnostics, School of Public Health, Xiamen University, Xiamen, Fujian, China

**Keywords:** gestational diabetes mellitus, prediction model, nomogram, early pregnancy, cholinesterase

## Abstract

Gestational diabetes mellitus (GDM) is one of the most common complications in pregnancy, impairing both maternal and fetal health in short and long term. As early interventions are considered desirable to prevent GDM, this study aims to develop a simple-to-use nomogram based on multiple common risk factors from electronic medical health records (EMHRs). A total of 924 pregnant women whose EMHRs were available at Peking University International Hospital from January 2022 to October 2022 were included. Clinical demographics and routine laboratory analysis parameters at 8-12 weeks of gestation were collected. A novel nomogram was established based on the outcomes of multivariate logistic regression. The nomogram demonstrated powerful discrimination (the area under the receiver operating characteristic curve = 0.7542), acceptable agreement (Hosmer-Lemeshow test, *P* = 0.3214) and favorable clinical utility. The C-statistics of 10-Fold cross validation, Leave one out cross validation and Bootstrap were 0.7411, 0.7357 and 0.7318, respectively, indicating the stability of the nomogram. A novel nomogram based on easily-accessible parameters was developed to predict GDM in early pregnancy, which may provide a paradigm for repurposing clinical data and benefit the clinical management of GDM. There is a need for prospective multi-center studies to validate the nomogram before employing the nomogram in real-world clinical practice.

## 1 Introduction

Gestational diabetes mellitus (GDM) is a common chronic complication defined as glucose intolerance with onset or first detection in pregnancy (1), affects the health of millions of pregnant women worldwide (2), and its prevalence has been up to 14.8% in China (3). The pathogenesis of GDM in pregnant women primarily involves insulin resistance and β-cell defects, characterized by the inability of pancreatic β-cells to adequately respond to the increased insulin demands of pregnancy, contributing to varying degrees of hyperglycemia (4, 5). In addition, increased circulating placental-related insulin antagonists, including growth hormone, corticotrophin-releasing hormone, human placental lactogen, prolactin, estrogen, and insulinase, cause a reduction in insulin sensitivity of approximately 50-60% in late pregnancy, leading to a progressive increase in insulin resistance (5, 6). The hyperglycemia is closely associated with short- and long-term negative health of mother and offspring (6). Pregnant women with GDM have an increased risk of preeclampsia and cesarean section in the short term (7), and a higher risk of co-morbidities including recurrence of GDM and type 2 diabetes mellitus (T2DM) in the long term (8, 9). Similarly, the offspring of women with GDM are at high risk of preterm birth and neonatal hypoglycemia in the short term (10), and T2DM and cardiovascular diseases in the long term (11, 12).

It was reported that the typical diagnosis of GDM at 24-28 weeks of gestation made it too late for the reversal of adverse effects mainly because of the short window left for glycemic control (13). The poor results of insulin therapy and diet-exercise interventions underlying the evidence that fetal development has already occurred before the oral glucose tolerance test (OGTT) (13, 14). While the therapies after OGTT were limited, early identification of GDM and interventions that subsequently initiated in the first or second trimester were considered desirable as these might be able to prevent GDM and reduce the risk of its associated co-morbidities (15). Given that the increased prevalence of GDM may occur along with the implementation of three-child policy in China, it is thus of great importance to predict and identify GDM in the early pregnancy for the prevention of GDM.

Publications show that risk factors for GDM include ethnicity and maternal factors such as older age, high parity, overweight and obesity, excessive weight gain in the index pregnancy, short stature, polycystic ovarian syndrome, history of diabetes mellitus in first-degree relatives, a history of poor pregnancy outcome (abortion, fetal loss), macrosomia in previous and/or index pregnancy, GDM in a previous pregnancy, pre-eclampsia, and multifetal pregnancy (16). As for the early prediction of GDM, despite much progress in identifying novel GDM biomarkers including exosomes, microRNA, and plasma fatty acid-binding protein 4 (17–19), however, lack of the availability in clinical practice leads to their limited application. To address the problems mentioned above, predicting GDM based on common risk factors has been proposed, yet most of them usually use a single variable or biomarker, resulting in the disadvantage of univariate approaches, that is, modest predictive accuracy (20). The strategy of establishing a prediction model using multiple common risk factors in early pregnancy provides a novel perspective in the economic and accurate prediction of GDM (21), as well as reducing unnecessary burdens on women without GDM by identifying low-risk women since OGTT requires multiple blood collections and is cumbersome to apply (22).

Electronic medical health records (EMHRs) are easily accessible clinical data resources (23) and have therefore been highlighted as invaluable for common data research (24) and feasible for construction of prediction models (23, 25). Regrettably, few prediction models of GDM have hitherto been established and widely accepted in clinical practice (22, 26). Here, we aimed to screen the clinical demographic and routine laboratory analysis parameters from EMHRs at the early stage of pregnancy, and then select potential variables that relevant with GDM to establish an early, cost-effective, and accurate prediction nomogram of GDM, which may serve as a novel prevention approach for GDM.

## 2 Material and methods

### 2.1 Study population and sample size calculation

**Figure 1:**
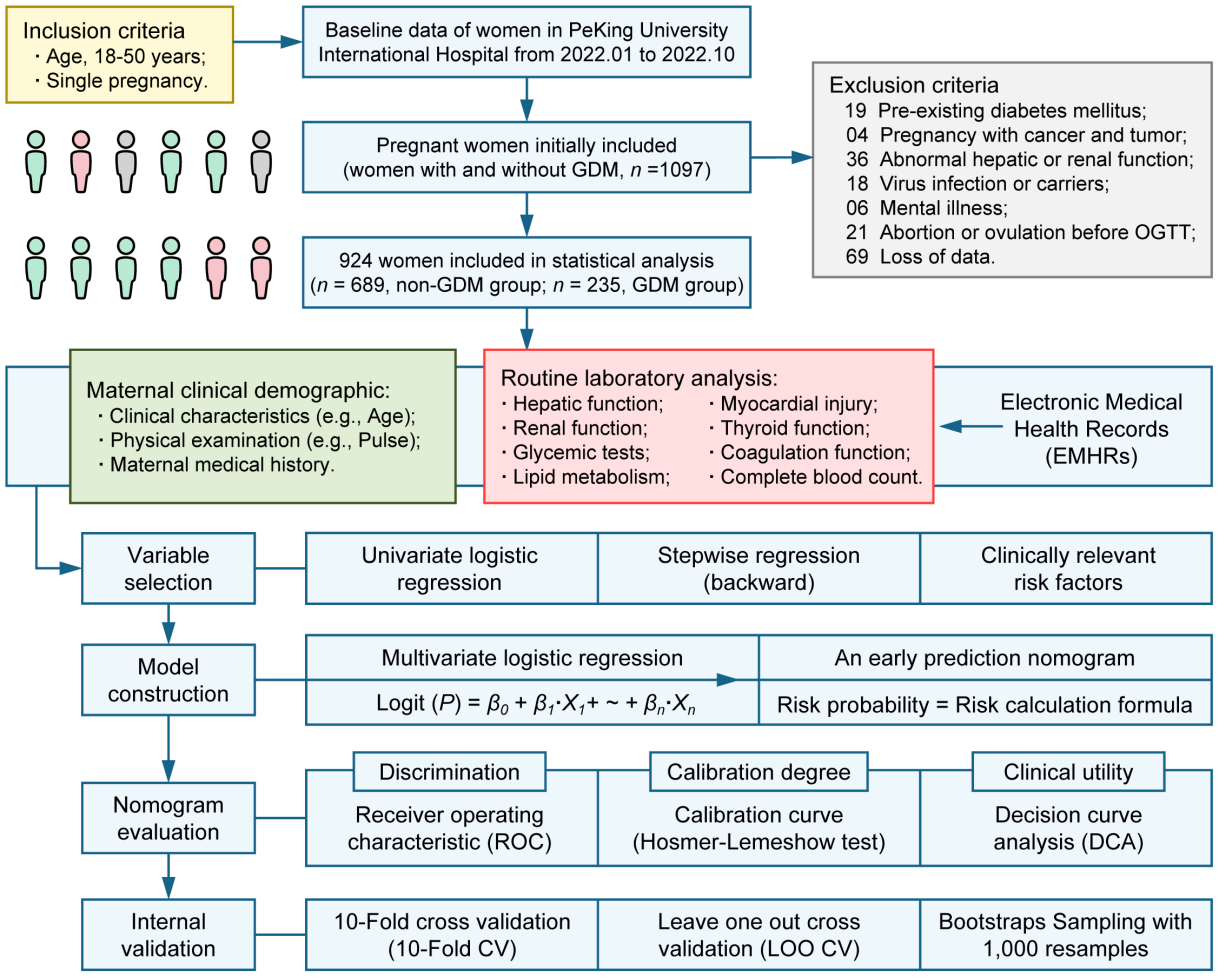
Study design.

This study included pregnant women for which electronic medical health record data were available at Peking University International Hospital from January 2022 to October 2022 (**Figure 1**). A total of 1097 women aged 18-50 years with a singleton pregnancy were initially included. We excluded samples based on the following criteria: pre-existing diabetes mellitus (*n* = 19); pregnancy combined with cancer and tumor (*n* = 4); abnormal hepatic function or renal function due to other diseases (*n* = 36); virus infection or carriers (*n* = 18); mental illness (*n* = 6); abortion or ovulation before OGTT (*n* = 21); loss of data (*n* = 69). This study was approved by the Medical Ethics Committee of Peking University International Hospital (No. 2016032).

According to the guideline for calculating sample size of the clinical prediction model with binary outcome, a target C-statistic, the number of predictors and the prevalence of GDM are the parameters required for sample size calculation (27). With a target C-statistic of 0.8, 12 candidate predictors and a GDM prevalence of 14.8% (3), the minimum sample size required for new model development is 664 participants (99 events) assuming 0.05 as acceptable difference between apparent and adjusted R^2^. Therefore, the sample size of this study met the requirements for developing a clinical prediction model with sufficient accuracy.

### 2.2 The diagnosis of gestational diabetes mellitus

OGTT was performed for the diagnosis of GDM at 24-28 weeks based on the International Association of Diabetes and Pregnancy Study Groups (IADPSG) guidelines (28). Pregnant women were admitted to Peking University International Hospital after 8-12 hours of fasting at 24-28 weeks of gestation. 75g of glucose powder would be dissolved in warm water and was then taken orally by pregnant women within 5 minutes. GDM cases would be diagnosed if their one or more values met the following criteria: 0-hour plasma glucose ≥ 5.1 mmol/L; 1-hour plasma glucose ≥ 10.0 mmol/L; 2-hour plasma glucose ≥ 8.0 mmol/L.

### 2.3 Clinical demographics parameters of pregnant women

Clinical demographics parameters at 8-12 weeks of gestation were obtained from maternal EMHRs (**Table 1**). (1) Basic characteristics including age, parity and gravidity, etc. in maternal the questionnaire data were recorded in their pregnancy files. (2) Physical measurement: systolic blood pressure (SBP), diastolic blood pressure (DBP) and pulse were measured. (3) Maternal medical history: history of abnormal pregnancy, cesarean delivery, GDM, and macrosomia were collected.

**Table 1:** Clinical demographics parameters of women with and without GDM in the first trimester.

Parameters	Total (*n* = 924)	GDM (*n* = 235)	non-GDM (*n* = 689)	*P* value
Maternal age (years)	32.31 ± 3.74	33.50 ± 3.93	31.90 ± 3.59	0.000
Systolic blood pressure (mmHg)	110.80 ± 10.77	112.78 ± 10.55	110.12 ± 10.77	0.001
Diastolic blood pressure (mmHg)	64.68 ± 8.45	65.72 ± 8.39	64.33 ± 8.45	0.029
Pulse (times/min)	77.46 ± 10.18	78.80 ± 10.22	77.01 ± 10.14	0.019
Smoker or Passive smoking *n* (%)	159 (17.2)	32 (13.6)	127 (18.4)	0.112
Method of conception *n* (%)				0.209
Ovulation drugs induction or *in vitro* fertilization	64 (6.9)	21 (8.9)	43 (6.2)	
natural pregnancy	860 (93.1)	214 (91.1)	646 (93.8)	
Gravidity *n* (%)				0.012
G = 0	488 (52.8)	107 (45.5)	381 (55.3)	
G ≥ 1	436 (47.2)	128 (54.5)	308 (44.7)	
Parity *n* (%)				0.063
P = 0, Nulliparous	674 (72.9)	160 (68.1)	514 (74.6)	
P ≥ 1, Multipara	250 (27.1)	75 (31.9)	175 (25.4)	
History of abnormal pregnancy *n* (%)	289 (31.3)	85 (36.2)	204 (29.6)	0.073
History of cesarean delivery *n* (%)	34 (3.7)	8 (3.4)	26 (3.8)	0.953
History of GDM *n* (%)	30 (3.2)	22 (9.4)	8 (1.2)	0.000
History of macrosomia *n* (%)	18 (1.9)	5 (2.1)	13 (1.9)	0.788
OGTT at 24-28 gestational weeks				
0-h blood glucose (mmol/L)	4.56 ± 0.43	4.64 ± 0.46	4.54 ± 0.41	0.000
1-h blood glucose (mmol/L)	7.96 ± 1.69	8.52 ± 1.76	7.76 ± 1.62	0.000
2-h blood glucose (mmol/L)	6.78 ± 1.32	7.16 ± 1.45	6.65 ± 1.25	0.000

Continuous data are expressed as means ± (standard deviation, SD) or median (interquartile range, IQR). Categorical data are expressed as n (percentages, %). *P* values (two-tailed) were calculated using Student’s t-test, Chi-square test or Fisher’s exact test. Significant at the *P* < 0.05 level.

### 2.4 Routine laboratory analysis parameters of pregnant women

Venous blood samples at 8-12 weeks of gestation were collected for routine laboratory analysis (**Table 2**). Laboratory measurements including: (1) hepatic function tests (Beckman AU 5800, USA); (2) renal function tests (Beckman AU 5800, USA); (3) glycemic tests (Beckman AU 5800, USA, glycated hemoglobin was measured using glycosylated hemoglobin analyzer); (4) lipid metabolism tests (Beckman AU 5800, USA); (5) myocardial injury tests (Beckman AU 5800, USA); (6) thyroid function tests (Roche Cobas E601, Switzerland), (7) coagulation function tests (Werfen ACL TOP 700, USA); (8) complete blood count (Sysmex XN-1000, Japan).

**Table 2:** Routine laboratory analysis parameters of women with and without GDM in the first trimester.

Parameters	Total (*n* = 924)	GDM (*n* = 235)	non-GDM (*n* = 689)	*P* value
Hepatic function				
Alanine aminotransferase, ALT (U/L)	13.0 (10.0-18.0)	14.0 (11.0-21.0)	13.0 (10-17.0)	0.000
Aspartic aminotransferase (U/L)	17.35 ± 5.85	17.43 ± 5.24	17.32 ± 6.05	0.792
Gamma glutamyltransferase (U/L)	14.0 (12.0-19.0)	16.0 (13.0-22.0)	14.0 (11.0-18.0)	0.000
Alkaline phosphatase, ALP (U/L)	52.0 (44.0-61.0)	54.0 (46.0-63.0)	51.0 (43.0-59.0)	0.011
Total bilirubin (μmol/L)	10.3 (8.5-13.2)	9.6 (8.05-12.05)	10.6 (8.7-13.4)	0.001
Direct bilirubin (μmol/L)	3.50 (2.80-4.60)	3.40 (2.70-4.20)	3.60 (2.90-4.60)	0.001
Total protein (g/L)	71.69 ± 3.87	71.73 ± 3.92	71.67 ± 3.85	0.834
Prealbumin (mg/L)	243.13 ± 36.49	252.72 ± 36.43	239.86 ± 35.96	0.000
Albumin (g/L)	43.67 ± 2.57	43.75 ± 2.68	43.65 ± 2.54	0.592
Cholinesterase (U/L)	7232.5 (6439.2-8167.0)	7747.0 (6853.0-8586.5)	7071.0 (6281.0-7919.0)	0.000
Renal Function				
Creatinine (μmol/L)	51.80 ± 6.13	51.70 ± 5.95	51.83 ± 6.19	0.791
Urea (mmol/L)	3.04 (2.57-3.54)	3.03 (2.62-3.56)	3.04 (2.56-3.53)	0.367
Uric acid (μmol/L)	217 (190-252.25)	227 (199-266)	213 (188-247)	0.000
Beta2-microglobulin (mg/L)	1.00 ± 0.19	1.00 ± 0.19	1.00 ± 0.18	0.611
Cystatin C (mg/L)	0.56 ± 0.08	0.56 ± 0.09	0.56 ± 0.08	0.521
Estimated glomerular filtration rate (ml/min/1.73cm^2^)	122.8 ± 6.48	121.44 ± 6.16	122.70 ± 6.55	0.010
Glycemic tests				
Fasting plasma glucose (mmol/L)	4.85 ± 0.37	4.97 ± 0.40	4.80 ± 0.35	0.000
Glycated hemoglobin (%)	5.30 ± 0.27	5.39 ± 0.27	5.27 ± 0.26	0.000
Lipid Metabolism				
Total cholesterol (mmol/L)	4.07 (3.69-4.55)	4.29 (3.79-4.68)	4.01 (3.66-4.49)	0.000
Triglyceride (mmol/L)	0.85 (0.65-1.20)	1.06 (0.77-1.38)	0.82 (0.63-1.07)	0.000
High density lipoprotein cholesterol (mmol/L)	1.42 (1.24-1.61)	1.39 (1.21-1.55)	1.43 (1.26-1.63)	0.018
Low density lipoprotein cholesterol (mmol/L)	2.15 (1.83-2.54)	2.31 (1.94-2.73)	2.11 (1.80-2.47)	0.000
Apolipoprotein A1 (mg/dL)	157.57 ± 32.20	161.54 ± 34.53	156.21 ± 31.28	0.028
Apolipoprotein B (mg/dL)	70.75 ± 17.58	74.92 ± 16.59	69.32 ± 17.70	0.000
Lipoprotein (a) (mg/L)	74.0 (36.0-165.5)	78.0 (35.0-176.0)	73.0 (36.0-165.0)	0.852
Small dense low density lipoprotein cholesterol (mmol/L)	0.60 (0.47-0.78)	0.72 (0.52-0.88)	0.57 (0.46-0.73)	0.000
Myocardial Injury				
Lactate dehydrogenase (U/L)	142.98 ± 23.41	144.59 ± 22.77	142.43 ± 23.62	0.223
Alpha-hydroxybutyrate dehydrogenase (U/L)	94.42 ± 13.57	94.88 ± 13.30	94.27 ± 13.66	0.547
Creatine kinase (U/L)	46.0 (37.0-57.25)	49.0 (39.0-58.0)	45.0 (37.0-57.0)	0.096
Creatine kinase-MB (U/L)	9.00 (7.00-11.00)	9.00 (7.00-11.00)	9.00 (7.00-11.00)	0.346
Homocysteine (μmol/L)	7.04 ± 1.49	6.90 ± 1.27	7.08 ± 1.56	0.095
Highly sensitive c-reactive protein (mg/L)	1.15 (0.62-2.40)	1.66 (0.88-2.94)	1.04 (0.57-2.09)	0.000
Thyroid Function				
Thyroid stimulating hormone (μIU/L)	1.50 (0.95-2.24)	1.60 (1.03-2.44)	1.47 (0.91-2.11)	0.011
Thyroid hormone (nmol/L)	126.31 ± 26.92	126.29 ± 28.20	126.32 ± 26.49	0.988
Triiodothyronine thyroid gland (nmol/L)	2.07 ± 0.44	2.07 ± 0.46	2.07 ± 0.44	0.894
Free thyroid gland hormone (pmol/L)	17.49 ± 2.58	17.20 ± 2.58	17.59 ± 2.57	0.048
Free triiodothyronine thyroid gland (pmol/L)	4.87 ± 0.80	4.81 ± 0.66	4.88 ± 0.85	0.245
Coagulation Function				
D-dimer (ng/mL)	85.0 (53.0-139.0)	82.0 (54.0-146.5)	86.00 (53.0-133.0)	0.931
Activated partial thromboplastin time (s)	30.29 ± 2.57	29.80 ± 2.41	30.45 ± 2.60	0.001
Thrombin time (s)	13.86 ± 0.93	13.75 ± 0.92	13.89 ± 0.93	0.041
Prothrombin time (s)	11.58 ± 0.71	11.48 ± 0.72	11.62 ± 0.70	0.008
Fibrin degradation products (μg/mL)	0.89 (0.66-1.24)	0.89 (0.68-1.25)	0.89 (0.65-1.23)	0.890
Fibrinogen (mg/dL)	330.14 ± 48.40	340.73 ± 53.11	326.53 ± 46.17	0.000
Complete Blood Count				
Platelet count (×10^9^/L)	246 (213-282)	261 (223.5-288)	243 (208-276)	0.000
Red blood cell count (×10^12^/L)	4.33 ± 0.34	4.37 ± 0.37	4.32 ± 0.32	0.062
White blood cell count (×10^9^/L)	7.77 ± 1.94	8.14 ± 1.97	7.65 ± 1.92	0.001
Neutrophil count (×10^9^/L)	5.45 ± 1.66	5.78 ± 1.63	5.33 ± 1.65	0.000
Neutrophil percentage (%)	69.38 ± 6.29	70.52 ± 5.50	68.99 ± 6.49	0.001
Lymphocyte count (×10^9^/L)	1.73 (1.49-2.06)	1.75 (1.48-2.10)	1.73 (1.49-2.05)	0.470
Lymphocyte percentage (%)	23.0 (19.9-27.4)	22.2 (19.5-25.7)	23.2 (20.1-27.7)	0.006
Monocyte count (×10^9^/L)	0.38 (0.32-0.47)	0.39 (0.33-0.47)	0.38 (0.31-0.47)	0.385
Monocyte percentage (%)	5.10 (4.38-6.00)	4.90 (4.20-5.70)	5.10 (4.40-6.00)	0.013
Eosinophil count (×10^9^/L)	0.06 (0.04-0.11)	0.07 (0.04-0.11)	0.06 (0.04-0.11)	0.505
Eosinophil percentage (%)	0.80 (0.50-1.40)	0.80 (0.50-1.40)	0.80 (0.50-1.50)	0.622
Basophil count (×10^9^/L)	0.40 (0.30-0.50)	0.40 (0.30-0.50)	0.40 (0.30-0.50)	0.132
Basophil percentage (%)	0.03 (0.02-0.04)	0.03 (0.02-0.04)	0.03 (0.02-0.04)	0.867

Continuous data are expressed as means ± SD or median (IQR). *P* values (two-tailed) were calculated using Student’s t-test or Wilcoxon rank sum test. Significant at the *P* < 0.05 level.

### 2.5 Statistical analysis

Continuous data with a normal distribution were expressed as mean ± (standard deviation, SD) and non-normal data as median (interquartile range, IQR). Categorical data were expressed as *n* (percentages, %). *P* values (two-tailed) were calculated using Student’s *t*-test or Wilcoxon rank sum test for continuous data. Chi-square test or Fisher’s exact test were used for calculating the *P* values (two-tailed) of categorical data. For variable selection, univariate logistic regression was used to screen candidate variables and stepwise regression (backward elimination) based on Akaike Information Criterion (AIC) was then used for further variable selection. Besides, clinically relevant risk factors were also included. For model construction, a novel nomogram based on the outcomes of multivariate logistic regression analysis was established using statistically significant predictors in the multivariate logistic regression model. For nomogram evaluation, receiver operating characteristic (ROC) analysis was performed and the area under the receiver operating characteristic curve (AUC, C-statistics) was used to evaluate the discrimination of the nomogram. We evaluated the calibration degree of the nomogram based on the agreement between predicted probability and observed probability, in addition, The Hosmer-Lemeshow test was used to assess the goodness of fit of the nomogram. The clinical utility of the nomogram was evaluated by the introduction of decision curve analysis (DCA). For internal validation, 10-Fold cross validation (10-Fold CV), Leave one out cross validation (LOO CV) and Bootstraps Sampling with 1, 000 resamples were used to evaluate the stability of the nomogram (**Figure 1**).

All statistical data analysis were performed using R Studio software (version 4.2.1) or GraphPad Prism software (version 9.5.0). Significant at the *P* < 0.05 level (*P < 0.05, **P < 0.01, ***P < 0.001).

## 3 Results

### 3.1 Baseline characteristics of women with and without GDM in the first trimester

In total, we included 235 instances of GDM pregnant women and 635 cases of non-GDM pregnant women in this study, the incidence of GDM was 25.43% (**Figure 1**). The candidate variables were collected for each pregnant women in the study. For clinical demographics parameters (**Table 1**), the average age, systolic blood pressure, diastolic blood pressure, and pulse of GDM women were significantly higher than non-GDM women (all *P* < 0.05). Despite GDM cases had higher gravidity ≥ 1 percentage and multipara rate than non-GDM cases, the difference of multipara rate between two groups didn’t reach statistically significance (*P* = 0.063). The percentage of women with previous GDM (Pre-GDM) was significantly higher in GDM group (9.4% vs. 1.2%, *P* < 0.001).

As for routine laboratory analysis parameters (**Table 2**), GDM cases had higher alanine aminotransferase (ALT), gamma glutamyltransferase (GGT), alkaline phosphatase (ALP), prealbumin (PA), cholinesterase (CHE) but lower total bilirubin (TBIL), direct bilirubin (DBIL) in hepatic function tests (all *P* < 0.05). In renal function tests, the levels of uric acid (UA) and estimated glomerular filtration rate (eGFR) were also higher in women with GDM (all *P* < 0.05). The fasting plasma glucose (FPG) and glycated hemoglobin (HbA1c) were both higher in GDM group than those in non-GDM group (all *P* < 0.001). In lipid metabolism tests, in addition to lower high density lipoprotein cholesterol (HDL-C) in GDM group, parameters including total cholesterol (TC), triglyceride (TG), low density lipoprotein cholesterol (LDL-C), apolipoprotein A1 (apo-A1), apolipoprotein B (apo-B) and small dense low density lipoprotein cholesterol (sdLDL-C) were higher in GDM group (all *P* < 0.05). Only the difference of highly sensitive c-reactive protein (hs-CRP) levels between GDM group and non-GDM group in the myocardial injury tests showed statistical significance (*P* < 0.001). In thyroid function tests, higher thyroid stimulating hormone (TSH) and lower free thyroid gland hormone (FT4) were observed in GDM group (all *P* < 0.05). In coagulation function tests, GDM cases had shorter activated partial thromboplastin time (APTT), thrombin time (TT), prothrombin time (PT), and increased fibrinogen (all *P* < 0.05). In complete blood count tests, increased platelet count (PLT), white blood cell count (WBC), neutrophil count (NC), neutrophil percentage (NP) and decreased lymphocyte percentage (LP), monocyte percentage (MP) were found in women with GDM (all *P* < 0.05).

### 3.2 Predictors setting for multivariate logistic regression model

**Figure 2:**
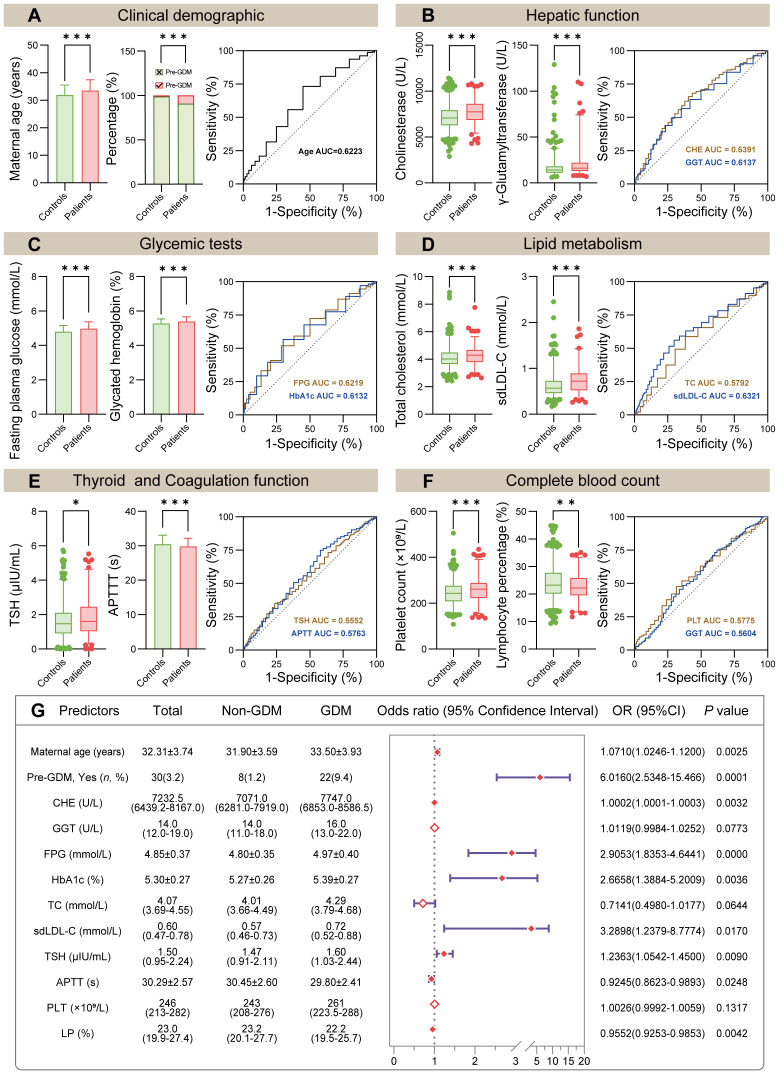
Comparison of initially selected predictors and model construction. **(A–F)** Comparison of 12 selected predictors between non-GDM group and GDM group (left panel). The ROC curve showed the AUC of indicated predictors (right panel, the detailed ROC analysis shown in **Table 3**). **(G)** Forest plot of multivariate logistic regression analysis based on the 12 predictors mentioned above. *Abbreviations:* Pre-GDM, previous GDM; CHE, cholinesterase; GGT, gamma glutamyltransferase; FPG, fasting plasma glucose; HbA1c, glycated hemoglobin; TC, total cholesterol; sdLDL-C, small dense low density lipoprotein cholesterol; TSH, thyroid stimulating hormone; APTT, activated partial thromboplastin time; PLT, platelet count; LP, lymphocyte percentage. In **(A–F)**, *P* values (two-tailed) were calculated using Student’s *t*-test, Wilcoxon rank sum test or Chi-square test (*P < 0.05, **P < 0.01, ***P < 0.001).

Initially, univariate logistic regression was performed for all clinical demographics and routine laboratory analysis parameters to screen potential GDM-related variables. With 34 statistically significant variables in the univariate logistic regression analysis, ROC analysis was simultaneously performed using GraphPad Prism to get a glimpse of the diagnostic value of these variables (**Table 3**). CHE, TG and sdLDL-C were the markers with the highest AUC among these variables. The AUC of each variable was lower than 0.7 indicating that the diagnostic value of single variable was limited. Stepwise regression (backward) was then used for further variable selection. Additionally, clinically relevant risk factors were included. The 12 selected predictors were as follows: Maternal age (33.50 vs. 31.90, *P* < 0.001), Pre-GDM (9.4% vs. 1.2%, *P* < 0.001), CHE (median, 7747.0 vs. 7071.0, *P* < 0.001), GGT (median, 16. vs. 14.0, *P* < 0.001), FPG (4.97 vs. 4.80, *P* < 0.001), HbA1c (5.39 vs. 5.27, *P* < 0.001), TC (median, 4.29 vs. 4.07, *P* < 0.001), sdLDL-C (median, 0.72 vs. 0.57, *P* < 0.001), TSH (median, 1.60 vs. 1.47, *P* < 0.05), APTT (29.80 vs. 30.45, *P* < 0.001), PLT (median, 261 vs. 243, *P* < 0.001), LP (median, 22.2% vs. 23.2%, *P* < 0.01) (**Figures 2A–F**, left panel). ROC analysis was performed for the continuous variables among these predictors (**Figures 2A–F**, right panel), and all predictors had limited diagnostic value when applied individually.

**Table 3:** Results of univariate logistic regression and ROC analysis for the statistically significant variables.

Parameters	OR (95% CI)	*P* value	Youden index	Cut-off	AUC	Sen (%)	Spe (%)	PPV (%)	NPV (%)
Age (years)	1.1184 (1.0751-1.1644)	0.0000	0.2109	31.50	0.6223	73.19	47.9	32.39	83.97
SBP (mmHg)	1.0235 (1.0094-1.0381)	0.0011	0.1215	110.5	0.5711	57.87	54.28	30.15	79.07
DBP (mmHg)	1.0196 (1.0019-1.0376)	0.0297	0.0861	65.50	0.5501	48.09	60.52	29.35	77.37
Pulse (times/min)	1.0173 (1.0027-1.0321)	0.0198	0.1337	74.50	0.5561	68.09	45.28	29.80	80.62
Gravidity, G≥1	1.4798 (1.0997-1.9945)	0.0098	—	—	—	—	—	—	—
Pre-GDM, yes	8.7923 (4.0100-21.311)	0.0000	—	—	—	—	—	—	—
CHE (U/L)	1.0004 (1.0002-1.0005)	0.0000	0.2433	7318	0.6391	65.11	59.22	35.26	83.27
GGT (U/L)	1.0220 (1.0102-1.0346)	0.0003	0.1913	14.50	0.6137	63.40	55.73	32.82	81.70
PA (mg/L)	1.0096 (1.0055-1.0138)	0.0000	0.1734	249.5	0.6038	53.19	64.15	33.60	80.07
TBIL (μmol/L)	0.9534 (0.9162-0.9896)	0.0150	0.1334	9.650	0.5702	50.64	62.70	31.65	78.83
DBIL (μmol/L)	0.8566 (0.7660-0.9517)	0.0051	0.1141	3.750	0.5732	64.68	46.73	29.29	79.50
UA (μmol/L)	1.0051 (1.0022-1.0081)	0.0007	0.1431	219.5	0.5825	58.72	55.59	31.08	79.79
eGFR (ml/min/1.73cm^2^)	0.9706 (0.9487-0.9929)	0.0102	0.0991	125.0	0.5563	73.19	36.72	28.29	80.06
FPG (mmol/L)	3.4848 (2.3079-5.3167)	0.0000	0.1911	4.950	0.6219	51.91	67.20	35.06	80.38
HbA1c (%)	5.5196 (3.0569-10.132)	0.0000	0.1901	5.350	0.6132	56.60	62.41	33.93	80.83
TC (mmol/L)	1.3245 (1.0884-1.6137)	0.0050	0.1713	4.195	0.5792	55.74	61.39	32.99	80.26
TG (mmol/L)	2.2817 (1.7492-3.0107)	0.0000	0.2812	1.005	0.6526	57.87	70.25	39.88	83.02
LDL-C (mmol/L)	1.5102 (1.1721-1.9487)	0.0014	0.1772	2.345	0.5895	49.36	68.36	34.73	79.83
sdLDL-C (mmol/L)	4.6545 (2.7106-8.0942)	0.0000	0.2528	0.685	0.6321	55.32	69.96	38.58	82.11
apo-A1 (mg/dL)	1.0049 (1.0005-1.0094)	0.0293	0.0917	144.5	0.5439	67.66	41.51	28.29	79.01
apo-B (mg/dL)	1.0176 (1.0092-1.0262)	0.0000	0.2113	74.50	0.6130	52.77	68.36	36.26	80.93
hs-CRP (mg/L)	1.0835 (1.0363-1.1395)	0.0010	0.1858	0.885	0.6232	74.89	43.69	31.21	83.61
TSH (μIU/L)	1.2292 (1.0650-1.4176)	0.0046	0.1080	2.145	0.5552	34.89	75.91	33.06	77.37
FT4 (pmol/L)	0.9419 (0.8868-0.9989)	0.0486	0.0957	16.15	0.5440	37.87	71.70	31.34	77.19
APTT (s)	0.9005 (0.8465-0.9564)	0.0008	0.1567	30.95	0.5763	74.89	40.78	30.13	82.64
PT (s)	0.7450 (0.5972-0.9240)	0.0081	0.1327	11.55	0.5626	60.00	53.27	30.46	79.61
TT (s)	0.8413 (0.7114-0.9910)	0.0410	0.0556	13.85	0.5378	54.04	51.52	27.55	76.67
FIB (mg/dL)	1.0059 (1.0029-1.0090)	0.0001	0.1216	358.0	0.5748	32.77	79.39	35.16	77.59
PLT (×10^9^/L)	1.0052 (1.0023-1.0080)	0.0004	0.1523	260.5	0.5775	50.64	64.59	32.79	79.32
WBC (×10^9^/L)	1.1333 (1.0518-1.2228)	0.0011	0.1466	6.930	0.5727	74.89	39.77	29.78	82.28
NC (×10^9^/L)	1.1695 (1.0712-1.2794)	0.0005	0.1748	4.905	0.5818	71.91	45.57	31.06	82.63
NP (%)	1.0413 (1.0162-1.0678)	0.0013	0.1428	67.85	0.5689	72.77	41.51	29.79	81.72
LP (%)	0.9604 (0.9339-0.9869)	0.0041	0.1264	25.55	0.5604	74.47	38.17	29.12	81.42
MP (%)	0.8714 (0.7715-0.9793)	0.0235	0.0858	4.850	0.5540	49.36	59.22	29.22	77.42

Sen, sensitivity; Spe, specificity; PPV, positive predictive value; NPV, negative predictive value; SBP, systolic blood pressure; DBP, diastolic blood pressure; Pre-GDM, previous GDM; CHE, cholinesterase; GGT, gamma galactosyltransferase; PA, prealbumin; TBIL, total bilirubin; DBIL, direct bilirubin; UA, uric acid; eGFR, estimated glomerular filtration rate; FPG, fasting plasma glucose; HbA1c, glycated hemoglobin; TC, total cholesterol; TC, total cholesterol; TG, triglyceride; LDL-C, low density lipoprotein cholesterol; sdLDL-C, small dense low density lipoprotein cholesterol; Apo-A1, apolipoprotein A1; apo-B, apolipoprotein B; hs-CRP, highly sensitive c-reactive protein; TSH, thyroid stimulating hormone; FT4, free thyroid gland hormone; APTT, activated partial thromboplastin time. PT, prothrombin time; TT, thrombin time; FIB, fibrinogen; PLT, platelet count; WBC, white blood cell; NC, neutrophil count; LP, NP, neutrophil percentage; lymphocyte percentage; MP, monocyte percentage.

### 3.3 Establishment of a novel nomogram based on multivariate logistic regression model

**Figure 3:**
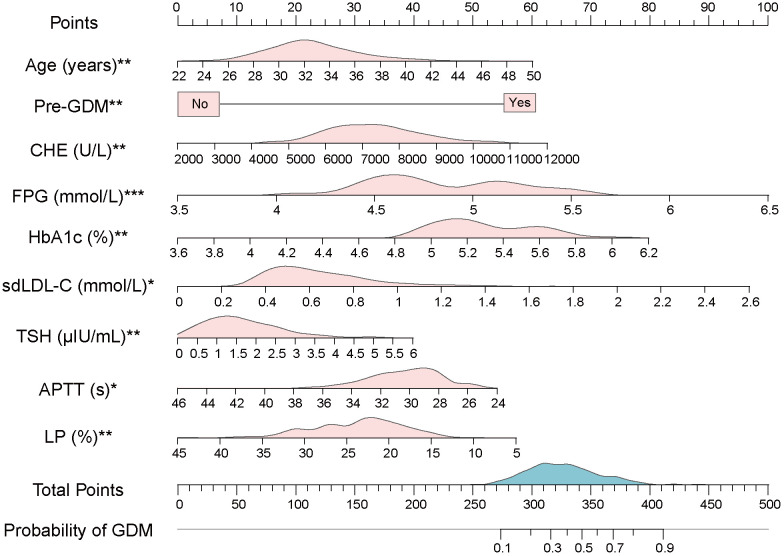
A novel nomogram based on multivariate logistic model. The value of each variable of pregnant women was placed on the corresponding points of the axis. The points on each row were aligned with the points on the top point scale to calculate the total points. The total score was then used to determine the risk probability of GDM. The pink area on the axis represents the distribution of the corresponding variable. The blue area on the axis shows the distribution of Total Points. *Abbreviations:* Pre-GDM, previous GDM; CHE, cholinesterase; FPG, fasting plasma glucose; HbA1c, glycated hemoglobin; sdLDL-C, small dense low density lipoprotein cholesterol; TSH, thyroid stimulating hormone; APTT, activated partial thromboplastin time; LP, lymphocyte percentage. Significant at the *P* < 0.05 level (*P < 0.05, **P < 0.01, ***P < 0.001).

Multivariate logistic regression model was constructed using 12 selected predictors. The final model equation was Logit (*P*) = -12.48 + 0.069 × Age (years) + 1.794 × Pre-GDM (yes vs. no) + 0.0002 × CHE (U/L) + 1.067 × FPG (mmol/L) + 0.9805 × HbA1c (%) + 1.191 × sdLDL-C (mmol/L) + 0.212 × TSH (μIU/L) + -0.079 × APTT (s) + -0.046 × LP (%). As shown in the forest plot, the odds ratio (OR) of 9 predictors and their 95% confidence interval (95% CI) were as follows: Age (OR 1.0710, 95% CI 1.0246-1.1200, *P* = 0.0025), Pre-GDM (yes vs. no, OR 6.0160, 95% CI 2.5348-15.466, *P* = 0.0001), CHE (OR 1.0002, 95% CI 1.0001-1.0003, *P* = 0.0032), FPG (OR 2.9053, 95% CI 1.8353-4.6441, *P* < 0.0001), HbA1c (OR 2.6658, 95% CI 1.3884-5.2009, *P* = 0.0036), sdLDL-C (OR 3.2898, 95% CI 1.2379-8.7774, *P* = 0.0170), TSH (OR 1.2363, 95% CI 1.0542-1.4500, *P* = 0.009), APTT (OR 0.9245, 95% CI 0.8623-0.9893, *P* = 0.0248), LP (OR 0.9552, 95% CI 0.9253-0.9853, *P* = 0.0042) (**Figure 2G**; **Table 4**). Based on the predictors that were statistically significant in the multivariate logistic regression model, a novel nomogram was established (**Figure 3**). The risk factors in our model were as follows, Age, Pre-GDM(yes), CHE, FPG, HbA1c, sdLDL-C, and TSH.

**Table 4:** Results of multivariate logistic regression analysis.

Predictors	*B* value	SEM	*Z* value	*P* value	OR	95% CI lower	95% CI higher
Age	0.069	0.023	3.027	0.002	1.071	1.025	1.120
Pre-GDM, yes	1.794	0.456	3.937	0.000	6.016	2.535	15.466
CHE	0.0002	0.000	2.948	0.003	1.0002	1.0001	1.0003
FPG	1.067	0.237	4.509	0.000	2.905	1.835	4.644
HbA1c	0.981	0.337	2.913	0.004	2.666	1.388	5.201
sdLDL-C	1.191	0.499	2.387	0.017	3.290	1.238	8.777
TSH	0.212	0.081	2.612	0.009	1.236	1.054	1.450
APTT	-0.079	0.035	-2.245	0.025	0.924	0.862	0.989
LP	-0.046	0.016	-2.861	0.004	0.955	0.925	0.985

Pre-GDM, previous GDM; CHE, cholinesterase; FPG, fasting plasma glucose; HbA1c, glycated hemoglobin; sdLDL-C, small dense low density lipoprotein cholesterol. TSH, thyroid stimulating hormone; APTT, activated partial thromboplastin time; LP, lymphocyte percentage. Significant at the *P* < 0.05 level.

**Figure 4:**
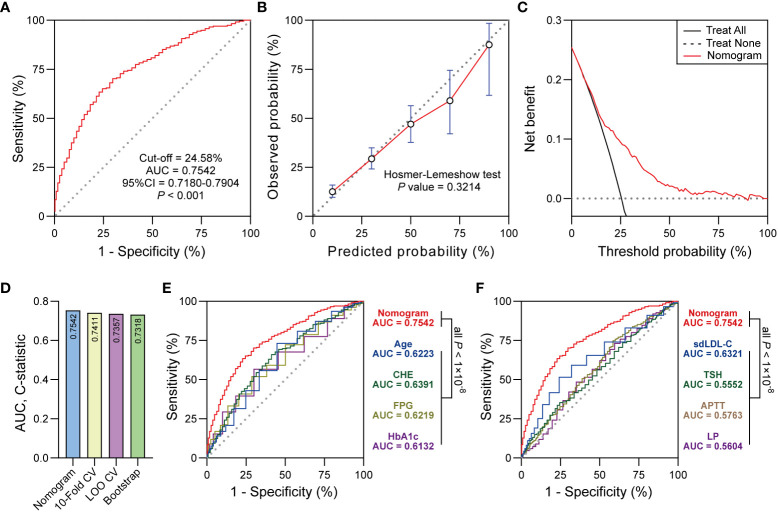
**(A)** ROC curve showed the discrimination of the nomogram. **(B)** The calibration curve showed the agreement between predicted probabilities and observed probabilities. **(C) **Decision curve analysis (DCA) showed the clinical utility of the nomogram. **(D)** 10-Fold CV, LOO CV and Bootstrap were performed for internal validation. **(E, F)** Delong test was used to compare the AUC between nomogram and predictors in the nomogram. Significant at the *P* < 0.05 level.

For the usage of the nomogram, pregnant women or clinicians only need to collect the value of each variable in the first-trimester pregnancy and place them on the points of corresponding axes. The points on each row were then aligned with the points on the top point scale to calculate the total points. The total points were finally used to determine the risk probability of GDM (**Figure 3**). For example, participant No. 1 in our data set had the age of 38 (years), no previous GDM, CHE of 8320 (U/L), FPG of 5.3 (mmol/L), HbA1c of 5.7 (%), sdLDL-C of 0.94 (mmol/L), TSH of 0.503 (μIU/L), APTT of 27 (s), and LP of 30.1 (%). To this participant, a total point of 304.57 and a risk probability of 27.71% were calculated using the established nomogram. This pregnant woman was therefore judged as GDM by the Cut-off value (24.58%, Cut-off value was shown in **Figure 4A**), which was in line with her actual state.

### 3.4 Nomogram evaluation and internal validation

The ROC curve derived from the nomogram achieved powerful discrimination (Cut-off = 24.58%, AUC = 0.7542, 95% CI 0.7180-0.7904, *P* < 0.001, **Figure 4A**). When the optimal threshold was 24.58%, the AUC of the nomogram was 0.7542 which was higher than any predictor in the nomogram according to the Delong test (**Figures 4E, F**). The sensitivity (Sen), specificity (Spe), positive predictive value (PPV), negative predictive value (NPV), and Youden Index of the nomogram were 70.21%, 70.54%, 44.84%, 87.41%, and 0.4075, respectively. The calibration curve indicated the acceptable agreement between the predicted values from the nomogram and the actual values from the observation (Hosmer-Lemeshow test, *P* = 0.3214, **Figure 4B**). In terms of the clinical utility, decision curve analysis showed the positive net benefit of the nomogram among majority of threshold probabilities (**Figure 4C**). Furthermore, with the C-statistics of 0.7411, 0.7357 and 0.7318 from 10-Fold CV, LOO CV and Bootstrap, respectively, the internal validation indicated the stability of the nomogram, which revealed the good predictive reproducibility of the nomogram among the modeling data set (**Figure 4D**).

## 4 Discussion

In recent years, the number of studies aimed at developing risk prediction models for GDM has increased along with the increased incidence of GDM. However, most of them used well-known risk factors for GDM as predictors of the models (e.g., age and pre-pregnancy body mass index) (29–31). This may result in the ineffective use of GDM-related serological indicators. Here, a novel nomogram integrating several common risk factors from EMHRs, in particular serological indicators, was established, which significantly improved the predictive performance compared to single markers. The predictors in the nomogram were found to be associated with previous clinical studies. With this inspiration, systematic description of these risk factors was as follows.

Previous studies have shown that severe insulin resistance increased with advancing age (32, 33). Pregnant women of advanced age faced with adverse changes in the number of insulin receptors and the insulin receptor signaling pathway, leading to insulin resistance and pancreatic β-cell defects, which increased the risk of GDM (33). Additionally, women with advanced age tend to be obese, and notably obesity might be the strongest known risk factor for GDM (34). Consistent with these, maternal age was observed to be a risk factor in our model (OR 1.0710, 95% CI 1.0246-1.1200). With the promotion of three-child policy in China, the number of elder pregnant women will increase rapidly in the future, and the incidence of GDM may rise as well. Thus, early prediction or screening of GDM should be performed for these pregnant women and timely interventions should be taken to reduce the incidence of GDM.

Of all the risk factors for recurrent GDM, Pre-GDM has been reported as a strong risk factor for up to 84% of GDM recurrences (8). Likewise, Pre-GDM had an adjusted OR of 6.0160 (95% CI 2.5348-15.466) in our multivariate logistic analysis. These are reminiscent of the recurrence of GDM may be associated with the genetics of GDM. Indeed, single-nucleotide polymorphisms (SNPs) in several genes were found to be involved in the regulation of insulin secretion and associated with the increased risk of GDM (35). This suggests that pregnant women with a history of GDM should be more aware of their blood glucose levels during pregnancy to avoid the recurrence of GDM.

The liver, an organ essential for maintaining glucose homeostasis and insulin resistance, plays an important role in the pathogenesis of metabolic diseases (36). Cholinesterase, which is synthesized in the liver and released into the blood to catalyze the hydrolysis of acetylcholine, etc., is a sensitive indicator of liver synthesis (37). A previous study showed that about 22% of patients with T2DM had abnormal liver function and increased CHE activity (38). Likewise, with an AUC of 0.6391 and an adjusted OR of 1.0002 in our ROC and multivariate logistic analysis, respectively, CHE was found to have elevated levels in GDM women. Most previous studies have focused on the association between CHE and T2DM, to our knowledge, the role of CHE in GDM remains unknown. Increased activity of CHE has been reported to be associated with insulin resistance in T2DM by regulating acetylcholine (39, 40). Specifically, in patients with T2DM combined with hyperlipidemia, the synthesis and transfer of fatty acids increased, leading to the upregulation of acylcholine (e.g., acetylcholine and butyrylcholine, both of which are substrates for serum). Thus, the elevated CHE levels may be caused by the substrate-induced increase in hepatic synthase (41). The increased activity of CHE in turn led to a decrease in acetylcholine, and the decrease in acetylcholine blocked the increase in intracellular Ca^2+^, negatively affecting the signaling pathway of blood glucose regulation after insulin binding to insulin receptors, thus causing insulin resistance (40, 41). It is worth noting that patients with diabetes mellitus may benefit from CHE inhibitors as these could reduce mortality and contribute to diabetes self-management (42). Given that GDM and T2DM share a similar pathogenesis, the mechanisms of CHE in developing and progressing GDM should be investigated in the future.

It was hypothesized that FPG levels in early pregnancy may not be able to accurately predict GDM before OGTT since FPG reflects blood glucose in a short term (43). However, it has also been suggested that FPG is simple and convenient to apply, and that high levels of FPG in the first trimester may predict GDM, although with limited accuracy (44). As for current study, FPG reached an AUC of 0.6219 when the threshold was 4.950 in our ROC analysis, and was found to be as a strong risk factor for GDM in our model (adjusted OR 2.9053, 95% CI 1.8353-4.6441). Notably, although the accuracy of single FPG was limited, FPG tests were advised for pregnant women to avoid pre-pregnancy diabetes mellitus (10). Besides, higher FPG levels in early pregnancy, even within the non-diabetic reference interval, increased the risk of adverse pregnancy outcomes (45). Unlike FPG, HbA1c indicates the average blood glucose levels over the last 8 to 12 weeks (46). It was suggested that high levels of HbA1c in the first trimester significantly increased the risk of GDM and was therefore useful for early prediction of GDM (47). Regrettably, the predictive accuracy of both FPG and HbA1c was limited when applied individually in our study. Encouragingly, our nomogram combining FPG and HbA1c with other risk factors has improved the prediction performance.

Pregnant women with higher lipid levels increased the risk of GDM, and the levels of sdLDL-C were slightly higher in women with GDM than in those without GDM (48, 49). It was reported that sdLDL-C was positively correlated with lipid levels and insulin resistance (50), suggesting that the increased risk of GDM may be associated with elevated levels of sdLDL-C. Indeed, higher sdLDL-C levels in early pregnancy were considered to have a significant predictive value for GDM (31). In line with these studies, our results confirmed that elevated sdLDL-C levels increased the risk of GDM (adjusted OR 3.2898, 95% CI 1.2379-8.7774). Thus, sdLDL-C may be an excellent marker of lipid metabolism for predicting GDM.

Thyroid function has a regulatory role in glucose metabolism and pancreatic function, characterized by the fact that the normal regulation of glucose can be disturbed by either deficient or excessive thyroid hormones (51). Indeed, it was suggested that higher TSH levels in early pregnancy, even within the normal reference range, increased the risk of GDM (52). Consistently, we found that elevated TSH levels were associated with the incidence of GDM (adjusted OR 1.2363, 95% CI 1.0542-1.4500). TSH has been reported to bind to receptors on adipocytes, leading to differentiation and proliferation of adipocytes (53), and translocated adiposity was strongly associated with the development of insulin resistance (54). This may explain the association between TSH and GDM found in our model and previous studies.

In terms of coagulation function, it has been reported that the endothelial damage caused by the hyperglycaemic state activated the internal coagulation system in women with GDM (55). It was noted that the coagulation function of pregnant women was enhanced from mid-pregnancy to reduce the risk of postpartum haemorrhage, known as physiological hypercoagulability (56). In addition, hypercoagulability was more likely to be found in women with GDM than in normal women during the same pregnancy (55). Confirming these, APTT, which reflects the state of the internal coagulation pathways, was shorter in women with GDM in our study (29.80 vs. 30.45, *P* < 0.001). It was thus suggested that, at least from the perspective of our study, differences in coagulation between GDM women and non-GDM women may occur in early pregnancy rather than in mid-pregnancy. Therefore, early monitoring of coagulation function indicators, especially APTT, may be useful in predicting GDM.

Although the etiology and pathogenesis of GDM are not fully explicit, studies have shown that inflammatory responses increased the risk of GDM (57, 58). As abnormal WBC indicate the inflammatory state, and elevated levels of WBC have been found in women with GDM, WBC may serve as a good predictive biomarker for GDM (57). In addition, elevated NC in the first trimester was found to be closely correlated with the development of maternal GDM and adverse pregnancy outcomes (59). Likewise, in our study, higher WBC and NC were observed in GDM women rather than in non-GDM women. Intriguingly, lymphocyte percentage, the predictor in our model as well as a type of peripheral WBC, was lower in women with GDM (median, 22.2% vs. 23.2%, *P* < 0.01) and had an adjusted OR of 0.9552 (95% CI 0.9253-0.9853). One likely scenario was that the increased NC was juxtaposed with the decreased lymphocyte count, leading to the increased neutrophil-to-lymphocyte ratio (NLR). Indeed, elevated NLR was found in GDM women due to the long-term chronic inflammatory state (60). Aberrant expression of inflammatory mediators promoted oxidative stress damage, leading to the apoptosis of peripheral blood lymphocytes, which may explain the lower LP in women with GDM in our study (61).

**Figure 5:**
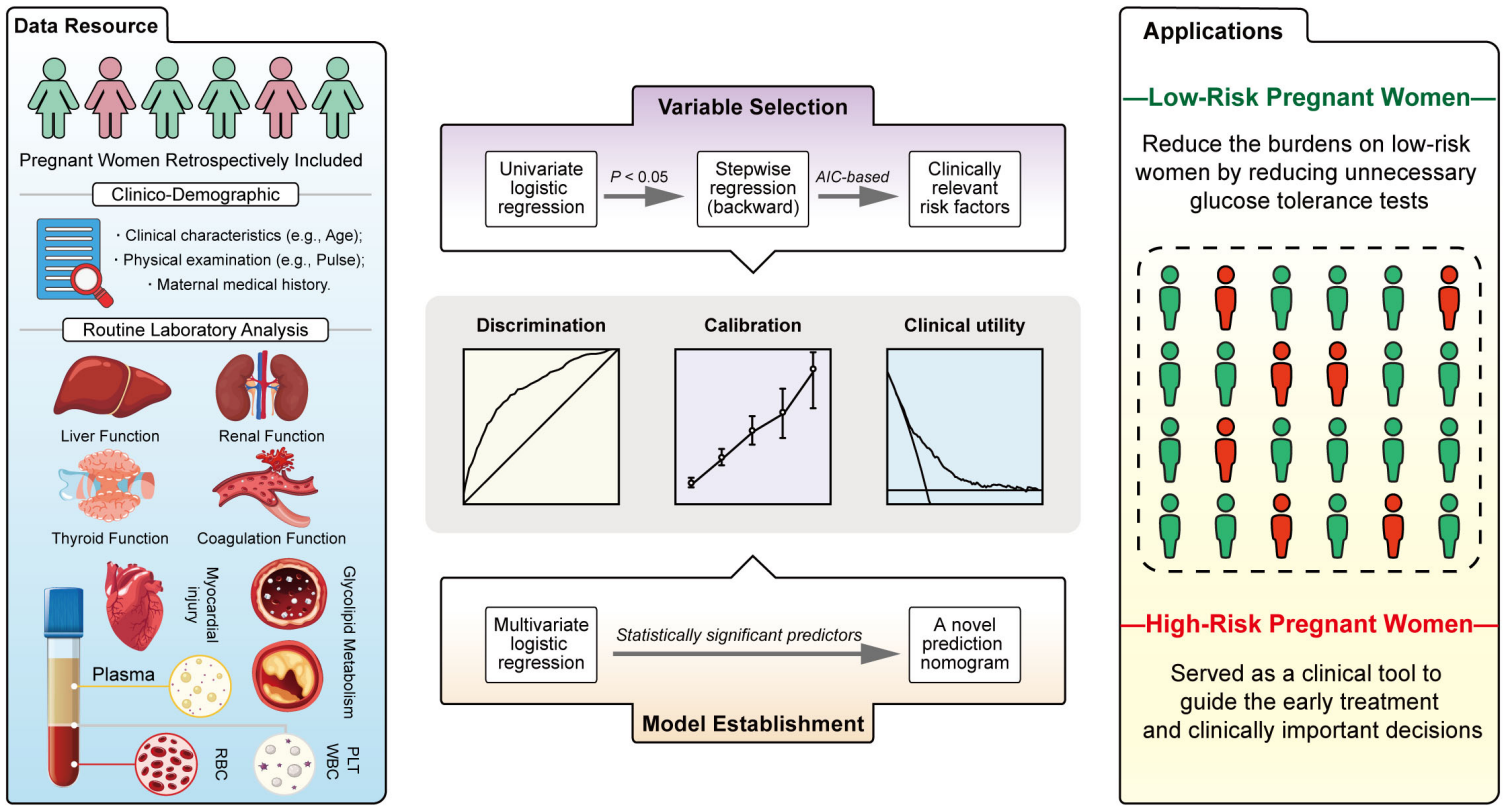
Summary of current study.

Taken together, by collecting clinical demographics and routine laboratory analysis parameters from EMHRs (**Figure 5**, blue panel) and screening potentially GDM-related variables (**Figure 5**, purple panel), this study established a simple-to-use nomogram for the prediction of GDM in the Chinese population (**Figure 5**, orange panel). The nomogram, which integrated several common risk factors, in particular serological indicators, demonstrated powerful discrimination, acceptable agreement and favorable clinical utility (**Figure 5**, grey panel). The 9 predictors in the nomogram are worthy of in-depth study, especially CHE, which may be closely associated with GDM. In this study, first-trimester CHE levels were found to be positively correlated with the risk of GDM after adjusting for confounders. We first developed a nomogram introducing CHE that could be used to accurately predict GDM. Our study may provide substantive clinical evidence to draw attention for exploring the association between CHE and GDM in the future. Introducing Age, Pre-GDM, CHE, FPG, HbA1c, sdLDL-C, TSH, APTT, LP, a nomogram based on these predictors might be used as a clinical tool to guide the early treatment for high-risk women and reduce the unnecessary burdens of glucose tolerance test on low-risk women (**Figure 5**, yellow panel). This is a clinically practical and cost-effective method for first-trimester screening for GDM, which is expected to be applied in district hospitals in the future, and is suitable for a wide range of people, despite special advanced detection equipment. Compared to previous models, the predictive variables included in this study cover several routine categories of laboratory test results, which are combined to objectively predict GDM.

However, there were also several limitations in our study. First, all data resource were collected from a single clinical center and the sample size of this study was relatively small. Second, this study established a nomogram for the prediction of GDM in the Chinese pregnant woman population. The nomogram needs external validation to prove its value. Since it is a prediction nomogram, the study needs to observe the follow-up development of positive individuals detected by the nomogram to assess the accuracy of the prediction. Further multi-center prospective studies are necessarily needed to evaluate and validate the nomogram prior to real-world clinical practice.

## 5 Conclusions

In summary, by integrating multiple common risk factors from EMHRs, this study developed a simple-to-use nomogram to predict GDM in the early pregnancy. In clinical practice, the results of the nomogram may allow early treatment for high-risk pregnant women and serve as a screening approach to avoid burdens of unnecessary OGTT on low-risk pregnant women.

## Data availability statement

All relevant data included in this study would not be publicly available due to privacy and ethical restrictions imposed by Peking University International Hospital. Requests to access the data can be directed to the corresponding authors.

## Ethics statement

This study was approved by the Medical Ethics Committee of Peking University International Hospital, the informed consent number was No. 2016032. The studies were conducted in accordance with the local legislation and institutional requirements. The human samples used in this study were acquired from a by- product of routine care or industry. Written informed consent for participation was not required from the participants or the participants’ legal guardians/next of kin in accordance with the national legislation and institutional requirements.

## Author contributions

Conceptualization: HZ, JD, JZ, HL. Supervision: JZ, HL, JD, XS, YS, LW. Analysis of most of the data: HZ. Visualization: HZ. Writing – original draft: HZ, JD, WZ. Writing – review & editing: JZ, HL, JD, WZ, HZ. All authors contributed to the article and approved the submitted version.
